# Foreword to the focus issue: cutting-edge chemistry and physics of soft materials

**DOI:** 10.1080/14686996.2026.2702249

**Published:** 2026-07-23

**Authors:** Shintaro Nakagawa, Koki Sano

**Affiliations:** a Faculty of Advanced Life Science, Hokkaido University, Sapporo, Japan; b Department of Chemistry and Materials, Faculty of Textile Science and Technology, Shinshu University, Ueda, Japan

Soft materials science is a relatively young research field in materials science. Human beings have used soft materials throughout history: natural materials such as fiber, fabric, leather, and wood, which we have relied on for a very long time, can all be considered soft materials that are fundamentally different from ‘hard’ materials like ceramics and metals. Nevertheless, the diverse and dynamic nature of soft materials has been well characterized and controlled only recently. One of the most significant milestones in soft materials science is the acceptance of the macromolecular hypothesis proposed by Staudinger almost a century ago. This concept established a new category of materials wherein small molecular units are connected to form large macromolecules that show unique properties.

Soft materials are diverse: combinations of distinct constituents, such as organic and inorganic components, natural and artificial materials, or polymers with different philicity, can lead to emergent functions. Soft materials are dynamic: they can change their shape and properties over time, and such changes can be induced by various stimuli (light, electric/magnetic fields, molecules, etc.) or can even be autonomously driven like living organisms. To make use of these fascinating aspects of soft materials, various efforts have been made to understand, characterize, design, and control their structures and properties across multiple length scales.

In 2024, we organized the 14th International Gel Symposium (Gel Symposium 2024) in Nago City, Okinawa, Japan, as an opportunity to share the latest progress in polymer networks and related soft materials. The symposium concluded with a major success, with over 100 invited speakers and many poster presenters gathered from all over the world. We gratefully acknowledge *Science and Technology of Advanced Materials* for kindly sponsoring a poster award. The success of Gel Symposium 2024 encouraged us to publish this Focus Issue to convey the dynamic progress in soft materials science to the broad readership of *Science and Technology of Advanced Materials*. Although the symposium mainly focused on gel materials, we broadened the scope of this Focus Issue to include a wider range of soft materials. This Focus Issue includes 2 review papers and 8 research articles contributed by the distinguished speakers at Gel Symposium 2024.

The Focus Issue showcases the recent progress in both the physical and chemical aspects of soft materials. On the physical side, one of the central challenges in soft materials science is to understand and predict mechanical responses across multiple length scales. From a theoretical perspective, Masubuchi et al. reviewed recent advances in molecular simulations of crosslinked polymer network rupture and discussed current challenges in bridging molecular-scale network structures and macroscopic mechanical failure [1]. From an experimental perspective, Katashima et al. investigated nonlinear viscoelastic behavior in a structurally well-defined transient polymer network through strain amplitude sweep measurements, providing insights into the molecular-level origins of nonlinearity in soft materials [2]. A representative example at the interface between physics and chemistry is self-organization in soft materials, which can be driven by the design and control of non-equilibrium conditions and molecular architectures. Okeyoshi et al. designed an open system with a tunable humidity gradient and demonstrated the universality of the meniscus-splitting phenomenon using a synthetic polymer solution, offering a versatile platform for studying dry/wet non-equilibrium self-organization [3]. In contrast, Patrickios et al. developed amphiphilic polymer conetworks composed of giant star block copolymers that self-organize into highly ordered nanophases with record-long repeat distances in both bulk and water, providing a promising strategy for constructing hierarchical soft materials through molecular design [4].

On the chemical side, this Focus Issue also highlights advances in stimuli-responsive soft materials based on molecular design. Miyata reviewed current progress and design principles of polymeric soft materials incorporating molecular recognition sites and discussed how molecular recognition events can be translated into structural and functional responses in hydrogels, particles, and interfaces [5]. Based on these design principles, Kawamura et al. developed bisphenol A-responsive microgels composed of hydrophilic polymer networks with molecular recognition sites, which exhibited rapid shrinkage in response to bisphenol A through molecular complex crosslinking [6]. Additionally, Imato and Ooyama et al. synthesized spiropyran-containing block copolymers and demonstrated that their microphase-separated environments can modulate the photoisomerization behavior of spiropyran photoswitches [7]. Extending the concept of stimuli-responsive soft materials, Enomoto and Yoshida et al. demonstrated that the autonomous motion of self-oscillating gels driven by the Belousov-Zhabotinsky (BZ) reaction can be modulated by electrochemical signaling, representing an important step toward the creation of artificial active matter with life-like behavior [8].

Beyond the physical and chemical aspects of soft materials, bioinspired design principles have also driven the development of functional hydrogels with hierarchical architectures. Nakajima et al. developed mechanically robust hydrogel composites by incorporating the hierarchically aligned structures of minimally processed fibrous agricultural products into a polymer network [9]. In addition, Ishikawa and Sakai et al. established a simple yet scalable strategy for deep hydroxyapatite mineralization using porous poly(ethylene glycol) sponge hydrogels, advancing the design of bioinspired soft-mineral composites for bone-mimetic scaffolds and regenerative materials [10].

Overall, these contributions highlight the progress in soft materials science from physical, chemical, and bioinspired perspectives through molecular design, structural hierarchy, macroscopic functionality, and non-equilibrium dynamics, expanding the frontiers of the field. We hope that this Focus Issue will stimulate further advances and new directions in soft materials science, and we look forward to scientific discussions and exchange at the upcoming Gel Symposium, to be held in February 2027 in Taipei, Taiwan.
